# Detection of invasive *Bartonella* infections with next-generation sequencing of microbial cell-free DNA

**DOI:** 10.1017/ash.2024.16

**Published:** 2024-02-12

**Authors:** Fernando H Centeno, Ahmed M. Hamdi, Todd M. Lasco, Mayar Al Mohajer

**Affiliations:** 1 Department of Medicine, Baylor College of Medicine, Houston, TX, USA; 2 Department of Pathology and Immunology, Baylor College of Medicine, Houston, TX, USA; 3 Baylor St. Luke’s Medical Center, Houston, TX, USA

## Abstract

We report 9 patients with invasive *Bartonella* infections, including 5 with endocarditis, who were diagnosed with microbial cell-free DNA next-generation sequencing and *Bartonella* serology studies. Diagnosis with plasma mcfDNA NGS enabled a faster clinical and laboratory diagnosis in 8 patients. Prompt diagnosis impacted antibiotic management in all 9 patients.

## Introduction

Disseminated *Bartonella* infections have a broad spectrum of clinical presentations, and the organism represents a particular diagnostic challenge due to its fastidious nature, long incubation times, and the limited sensitivity of *Bartonella* cultures.^1,2^ While more sensitive than cultures*, Bartonella* serology is not specific as cross-reactivity with *Coxiella* species has been reported and may adversely impact therapy and outcomes.^1^ Additionally, due to differing assay sensitivities, negative serology does not exclude active infection in the context of high clinical suspicion.^1^ Polymerase chain reaction (PCR) for *Bartonella* is sensitive and specific,^1^ but some commercial tests may only detect one or a limited number of species.

Particularly concerning with disseminated bartonellosis is pathogen seeding heart valves and other end organs. In the past 2 decades since its initial recognition as a causative organism for infective endocarditis, it has been increasingly demonstrated as a common pathogen in culture-negative endocarditis, representing 28% of cases in a French reference center.^3^ Management commonly includes the combination of doxycycline or a beta-lactam and gentamycin or rifampin,^4^ but more data is needed on these regimens.

Over the last decade, next-generation sequencing (NGS) of plasma microbial cell-free DNA (mcfDNA) has become an open-ended, noninvasive testing tool for diagnosing various pathogens. The mcfDNA NGS assay used in this study was the Karius Test™ by Karius (Redwood City, CA), a College of American Pathologists-accredited, Clinical Laboratory Improvement Amendments-certified mcfDNA NGS laboratory. This case series aimed to characterize the presentations of patients with disseminated bartonellosis and describe the role of mcfDNA NGS in diagnosis and tailoring adequate antimicrobial management.

## Method

This retrospective case series was performed at Baylor St. Luke’s Medical Center (BSLMC), a quaternary academic medical center, between 2017 and 2022. Patients with *Bartonella* serologies and *Bartonella* species concentrations at or above the mcfDNA NGS commercial threshold were included. These thresholds were established in a previously described cohort of 684 healthy adult patient sera used as healthy controls.^5^ This study was approved by the Baylor College of Medicine Institutional Review Board.

The electronic medical record for each patient was assessed for presenting symptoms and laboratory studies. The clinical turnaround time (TAT) and laboratory TAT were calculated for mcfDNA NGS, Bartonella serologies, and PCR. Clinical TAT was defined as the time from the physician’s order to reported results. Laboratory TAT was defined as the time from laboratory receipt of the patient sample by the send out laboratory to reported results. Modified Duke criteria were used to diagnose infective endocarditis.^6^

## Results

Thirty-eight patients with both mcfDNA NGS and *Bartonella* serology were reviewed.

Both tests were positive in 9 patients (8 with *B henselae* and 1 with *B vinsonii* speciated by mcfDNA NGS). There were no discrepancies found between them yielding a 100% accuracy for the mcfDNA NGS test compared with serology.

The median age for the 9 included patients was 45, and 7 of 9 patients were male. Their clinical characteristics and initial labs are shown in Table 1. Four patients had prosthetic valves. 2 had immunocompromising conditions, including 1 with HIV and 1 with a kidney transplant on immunosuppression. Two patients reported exposure to cats.

**Table 1.** Clinical features of patients infected with *Bartonella*

Case #	Reference interval	1	2	3	4	5	6	7	8	9
Age (years), gender	–	31, female	59, male	53, male	45, male	61, male	19, male	20, male	37, male	60, female
Associated conditions	–	Trisomy 21, pulmonary atresia with RV-PA catheter and LVOT obstruction.	History of resolved acute renal failure	History of aortic dissection with mechanical aortic valve repair	HIV (CD4 247), ESRD on peritoneal dialysis	Polycystic kidney disease requiring kidney transplant, complicated by cellular rejection; on immunosuppressive therapy	TOF s/p palliation with biventricular repair, RV-PA conduit replacement, and placement of a Melody valve	TOF and pulmonary atresia s/p BTT shunt, RV to PA conduit placement 4 years prior	Former PWID	None
Associated exposures	–	Dog and cat at home	Rancher of exotic animals	Work cleaning out horse stables	None found	Two cats at home	Dog and hamster at home	Two dogs at home	Unknown	None found
Initial suspected diagnosis	–	Bacterial, viral, or tickborne infection vs vasculitis	Malignancy, auto-immune disorder, *Brucella, Bartonella*	Gram-positive infective endocarditis, *Brucella, Bartonella,* or *Coxiella*	Coagulase-negative staphylococcal bacteremia	Viral infection	*Brucella*, *Bartonella* or *Coxiella*	Bacterial infective endocarditis	Bacterial infective endocarditis	Urosepsis
Fever >100.4°F	–	No	No	Yes	Yes	Yes	Yes	Yes	Yes	Yes
Shock	–	Yes	No	No	Yes	No	Yes	No	Yes	No
Initial labs										
WBC (k/µL)	4–10	5.3	4.8	3.1	8.2	4.43	6.1	6.9	4.6	7.5
Hemoglobin (g/dL)	12–15	12.3	6.0	6.0	5.0	13.8	7.8	8.4	9.0	12.0
Platelet count (k/µL)	150–450	22.4	115	134	142	155	116	204	101	281
BUN (mg/dL)	7–19	19	68	18	54	20	42	19	73	11
Creatinine (mg/dL)	0.6–1.3	3.3	5.1	0.8	15.2	1.6	3.1	1	7.9	0.8
C-Reactive protein (mg/Dl)	1	*Not done*	5.01	*Not done*	*Not done*	3.01	2.8	6.1	*Not done*	15.9
Procalcitonin (ng/mL)	<0.05	1.64	0.75	*Not done*	2.79	*Not done*	0.49	0.46	16.68	*Not done*
Lactate dehydrogenase (U/L)	115–220	448	269	277	*Not done*	*Not done*	247	218	*Not done*	318
Complement levels (mg/dL)	C3: 82-193 C4: 15-57	C3: 28 C4: 6	C3: 80 C4:13	*Not done*	*Not done*	*Not done*	*Not done*	*Not done*	C3: 39 C4: 6	*Not done*
PR3-ANCA (AI)	<1.0	2.6	6.1	<1.0	*Not done*	*Not done*	*Not done*	*Not done*	*Not done*	*Not done*
Fibrinogen (mg/dL)	225–434	79	186	*Not done*	*Not done*	*Not done*	208	*Not done*	202	550
Urinalysis										
Blood	Negative	Large	Large	Moderate	Large	Negative	Large	Large	Large	Negative
RBC (cells/HPF)	0–5	>182	237	1	600	3	51–100	21–50	>182	3
WBC (cells/HPF)	0–5	7	5	1	2	2	5–10	5–10	0	24
Protein (mg/dL)	Negative	50	30	Negative	100	+1 (semi-quantitative)	+2 (semi-quantitative)	+3 (semi-quantitative)	≥300	20
Microbiology										
Bartonella cultures	No growth	No growth × 4 blood cultures	*Not done*	*Not done*	*Not done*	Not done	*Not done*	*Not done*	*Not done*	*Not done*
Bartonella serology	IgM: 1:20	*B henselae:*	*B henselae:*	*B henselae:*	*B henselae:*	*B henselae:*	*B henselae:*	*B henselae:*	*B henselae:*	*B henselae:*
	IgG: 1:64	IgG: ≥1:1024	IgG ≥1:1024	IgG ≥1:1024	IgG ≥1:1024	IgG ≥1:2560	IgG ≥1:1024	IgG ≥1:1024	IgG ≥1:1024	IgG ≥1:1024
		IgM negative	IgM negative	IgM negative	IgM negative	IgM negative	IgM ≥ 1:256	IgM ≥ 1:256	IgM negative	IgM 1:160
		*B quintana:*	*B quintana:*	*B quintana:*	*B quintana:*	*B quintana:*	*B quintana:*	*B quintana:*	*B quintana:*	*B quintana:*
		IgG negative	IgG ≥1:1024	IgG negative	IgG negative	IgG ≥1:1280	IgG ≥1:512	IgG negative	IgG 1:64	IgG negative
		IgM negative	IgM negative	IgM negative	IgM negative	IgM negative	IgM negative	IgM ≥1:256	IgM negative	IgM 1:20
Bartonella PCR	250 copies/ml	*Not done*	Negative for *B henselae*, *B quintana*	*Not done*	*Not done*	*Not done*	*Not done*	*Not done*	*Not done*	Negative for *B henselae*, *B quintana*
mcfDNA *Bartonella* concentration and species	<10 MPM	Positive^a^ *B henselae*	26,651 *B vinsonii*	Positive^a^ *B henselae*	302 *B henselae*	Positive^a^ *B henselae*	33,034 *B henselae*	33, 430 *B henselae*	>316,000 *B henselae*	3,032 *B henselae*
Other mcfDNA NGS	–	None	None	None	EBV (557 MPM)^b^	Torque teno virus^a, b^	None	None	EBV (457 MPM)^b^	None
mcfDNA NGS clinical TAT (days)	–	3	2	2	2	5	7	2	2	3
mcfDNA NGS laboratory TAT (days)	–	1	1	1	1	1	3	1	1	1
Serology/PCR clinical TAT	–	6 (serology)	5 (serology) 2 (valve tissue PCR)	4 (serology)	4 (serology)	4 (serology)	3 (serology)	4 (serology)	4 (serology)	8 (serology) 4 (whole blood PCR)
Serology/PCR laboratory TAT	–	2 (serology)	2 (serology) 1 (valve tissue PCR)	2 (serology)	2 (serology)	4 (serology)	1 (serology)	4 (serology)	2 (serology)	8 (serology) 7 (whole blood PCR)
Imaging										
Trans-thoracic echocardiogram	–	Probable cleft mitral valve, otherwise unremarkable.	Calcified mass fixed on the posterior mitral valve leaflet.	Normal prosthetic aortic valve appearance and function	Mild aortic valve cusp thickening	*Not done*	Pulmonary valve thickening concerning for vegetation	New echobright densities in proximal (RV-PA) conduit with limited valve leaflet visualization	Severely enlarged left ventricle and large aortic valve echo-densities, largest 1.6 x 0.6 cm	No significant valvular heart disease
Trans-esophageal echocardiogram	–	*Not done*	Globular calcified density in posterior leaflet causing severe mitral regurgitation	No evidence of vegetations or endocarditis.	*Not done*	*Not done*	*Not done*	*Not done*	*Not done*	*Not done*
Clinical impact										
Modified Duke criteria	–	Possible	Definite	Definite	Rejected	Rejected	Definite	Definite	Definite	Rejected
Hospital stay (days)	–	10, 16^c^	25	21	9	Not hospitalized	42	6	46	16
ICU admission	–	Yes	Yes	Yes	No	No	Yes	No	Yes	No
Death	–	Yes	No	No	No	No	No	No	No	No
First positive diagnostic result and days to result	–	mcfDNA NGS (4 days)^d^	mcfDNA NGS (7 days)	mcfDNA NGS (7 days)	mcfDNA NGS (8 days)	mcfDNA NGS (21 days)	*Bartonella* serology (3 days)	mcfDNA NGS (not hospitalized)	mcfDNA NGS (8 days)	mcfDNA NGS (5 days)

Note. RV-PA: right ventricle-pulmonary artery; LVOT: left-ventricular outflow tract; PWID: person who injects drugs; ESRD: end-stage renal disease; TOF: tetralogy of Fallot; WBC: white blood cells; BUN: blood urea nitrogen; PR3-ANCA: proteinase 3-antineutrophil cytoplasmic antibodies; RBC: red blood cells; PCR: polymerase chain reaction; mcfDNA: microbial cell-free DNA; NGS: next-generation sequencing; MPM: molecules per microliter; TAT: turnaround time; ICU: intensive care unit; CMV: cytomegalovirus; EBV: Epstein-Barr virus; HHV: human herpesvirus; AFB: acid-fast bacillus; ANA: antinuclear antibody; ASO: antistreptolysin O; RF: rheumatoid factor; TPO: thyroid peroxidase; ANCA: antinuclear cytoplasmic antibody; TSH: thyroid-stimulating hormone.

a Some patients seen in 2017 received an earlier version of mcfDNA NGS studies that did not quantify the MPM of mcfDNA. Instead, the samples were tested with a negative buffer control.

b Epstein-Barr virus and torque teno virus were found in small concentrations in 2 patients, but were not considered clinically significant by treatment teams and may have been the result of mild viremia in the setting of a secondary source of critical illness.

c The patient was discharged after 10 days and was later readmitted for 16 days resulting in death.

d Tests for an organism not detectable by mcfDNA NGS assay.

Seven patients presented with fever, and four with septic shock. Eight had available transthoracic echocardiograms, two had valve thickening, and three had valvular masses or densities. Two patients had transesophageal echocardiograms, one of which was abnormal with a valvular density. Five patients met the modified Duke criteria for definite infective endocarditis, while one met the criteria for possible infective endocarditis, and three had neither.

Eight of nine patients were hospitalized for an average of 18.5 days (range 6–46 days). All patients had both positive mcfDNA NGS and *Bartonella* serology—for four, *Bartonella* was mentioned as part of the differential, and serologies were sent before mcfDNA NGS results, while for five, serologies were not sent until after mcfDNA NGS results. One had a valve tissue PCR, and another had a whole blood PCR that was negative for *Bartonella.* mcfDNA NGS was the first positive test for *Bartonella* in 8 patients, while serology was the first positive result in 1.

Serology had a median clinical TAT of 4 days (range 3–8) and a median laboratory TAT of 2 days (range 1–8). mcfDNA NGS had a median clinical TAT of 2 days (range 2–7) and a median laboratory TAT of 1 day (range 1–3**).** In the 8 cases where mcfDNA was the first to result, it impacted antimicrobial management. After diagnosis with *Bartonella* infection, 6 patients started doxycycline and rifampin, and 2 started doxycycline alone. One was already taking doxycycline for culture-negative endocarditis and had an adjustment in the gentamycin dose. Five discontinued unnecessary antibiotics.

## Discussion

Few studies exist on the incidence of disseminated bartonellosis or *Bartonella* endocarditis, potentially owing to the difficulty of diagnosing both with conventional laboratory equipment and their relatively recent description in the literature. Here, we present a case series of 9 patients with invasive bartonellosis, including 5 with endocarditis, diagnosed with mcfDNA NGS and Bartonella serologies. Similar to a study of French and Canadian patients with *Bartonella* endocarditis, we found a primarily male patient population with high rates of preexisting valvular disease, and we seldom identified zoonotic exposure.^7^

One of France’s largest reports of culture-negative endocarditis described 28% of cases with *Bartonella* as the etiology. However, 78% of these were associated with *Bartonella quintana,* which was not seen in our case series.^3^ By comparison, *Bartonella henselae* cases were more commonly associated with preexisting valvular disease and contact with cats^3^—both seen in this study. There is scarce literature on the proportion of invasive *Bartonella* infections associated with endocarditis, but a recent pediatric study showed 10 of 23 patients diagnosed with bartonellosis having endocarditis, consistent with the proportion seen in this series.^8^

The patients in this series presented with nonspecific symptoms and a broad differential. Culture-negative endocarditis was only suspected in three cases (Table 1). As a result, they received a battery of laboratory studies and were hospitalized for up to eight days before *Bartonella* was identified, and antimicrobial therapy was adjusted accordingly. These adjustments included starting targeted antibiotic coverage for Bartonella and discontinuing unnecessary antibiotics, including nephrotoxic treatment, in patients with compromised renal function. mcfDNA NGS could offer a significant advantage in diagnosing *Bartonella* infections, especially in those with critical presentations requiring prompt, directed antimicrobial therapy.

Echocardiogram findings in *Bartonella*-associated endocarditis can be variable and nonspecific. It is believed to cause larger vegetations and significant valvular damage, although this was not always observed in our limited case series.^9^

This study is limited by the small number of patients, the lack of a control group to compare clinical outcomes in patients diagnosed by conventional methods and the retrospective nature of the analysis. Additionally, clinical TATs may vary among different institutions, and laboratory TATs depend on the procedures of outside laboratories. Moreover, we did not perform a financial evaluation of this technique, which made us unable to assess its cost-effectiveness.

In conclusion, plasma mcfDNA NGS is an additional tool for diagnosing *Bartonella* species infections and may lead to improved patient outcomes. Still, larger studies with control groups are needed to evaluate the impact of these rapid diagnostics on clinical care, diagnostic stewardship, and overall cost.
